# m^6^A-Related lncRNAs Are Potential Prognostic Biomarkers of Cervical Cancer and Affect Immune Infiltration

**DOI:** 10.1155/2022/8700372

**Published:** 2022-04-11

**Authors:** Haixia Jia, Suhua Hao, Meiting Cao, Lifang Wang, Hua Bai, Wen Shui, Xiaotang Yang

**Affiliations:** ^1^Department of Prevention Care, Shanxi Province Cancer Hospital/Shanxi Hospital Affiliated to Cancer Hospital, Chinese Academy of Medical Sciences/Cancer Hospital Affiliated to Shanxi Medical University, Taiyuan, Shanxi, China; ^2^Department of Gynecology, Shanxi Province Cancer Hospital/Shanxi Hospital Affiliated to Cancer Hospital, Chinese Academy of Medical Sciences/Cancer Hospital Affiliated to Shanxi Medical University, Taiyuan, Shanxi, China; ^3^Department of Geratology, Shanxi Province Cancer Hospital/Shanxi Hospital Affiliated to Cancer Hospital, Chinese Academy of Medical Sciences/Cancer Hospital Affiliated to Shanxi Medical University, Taiyuan, Shanxi, China; ^4^Department of Gynecology and Obstetrics, Shanxi Bethune Hospital/Shanxi Academy of Medical Sciences, Tongji Shanxi Hospital/Third Hospital of Shanxi Medical University, Taiyuan, Shanxi, China; ^5^Department of ECG, Shanxi Province Cancer Hospital/Shanxi Hospital Affiliated to Cancer Hospital, Chinese Academy of Medical Sciences/Cancer Hospital Affiliated to Shanxi Medical University, Taiyuan, Shanxi, China; ^6^Department of Radiology, Shanxi Province Cancer Hospital/Shanxi Hospital Affiliated to Cancer Hospital, Chinese Academy of Medical Sciences/Cancer Hospital Affiliated to Shanxi Medical University, Taiyuan, Shanxi, China

## Abstract

The correlation of m^6^A-related lncRNAs with the prognosis and immune microenvironment of cervical cancer is not yet clear. In this study, we identified 7 m^6^A-related prognostic lncRNAs by Pearson correlation and univariate Cox regression analyses based on TCGA-cervical cancer dataset. Then, patients were divided into two clusters by consensus clustering based on the 7 m^6^A-related prognostic lncRNA expression. Cluster 1 was characterized by survival and stage disadvantage, enrichment of immunosuppressive and carcinogenic activation pathways. Besides, cluster 1 had higher immunosuppressive factor TGFbeta and lower immune cell infiltration compared with cluster 2. According to the expression of 7 m^6^A-related lncRNA, a 6-m^6^A-related lncRNA risk score model was established in the training set by LASSO regression analysis. The high-risk group had worse overall survival than the low-risk group. No matter in the training or validation sets, the m^6^A-related lncRNA risk score was an independent prognostic factor for overall survival. Meanwhile, we validated the independent prognostic value of risk score in the disease-specific survival and progression-free survival by multivariate Cox analysis. The high-risk group was characterized by higher TGFbeta and regulatory T cell and was rich in malignant pathways. Additionally, we also detected and compared the expression levels of four m^6^A-related prognostic lncRNA in 9 tumor samples and 9 normal tissues using quantitative real-time polymerase chain reaction assay. In conclusion, the novel m^6^A-related lncRNA risk score is a potential prognostic predictor of cervical cancer patients. These 6 m^6^A-related lncRNAs might serve as key mediators of the immune microenvironment and represent promising therapeutic targets for improving cervical cancer prognosis.

## 1. Introduction

The morbidity and mortality of cervical cancer rank fourth in female malignant tumors worldwide [1]. Approximately 604 thousand new cases and 342 thousand deaths from cervical cancer occurred in 2020 globally [1]. Among them, about 18% of new cases and 17% deaths occurred in China [2]. Patients with early-stage cervical cancer have an excellent prognosis, with a 5-year survival rate of more than 90% [3]. However, patients with recurrent and/or advanced cervical cancer have limited treatment options and poor prognosis, with a 5-year survival probability of 17% [4]. Several clinical features and molecular markers have been applied to predict the prognosis of cervical cancer patients, while these approaches are all limited to some extent. Therefore, it is indispensable to construct a new predictive model and identify new prognostic markers for cervical cancer.

Among more than 150 RNA modifications in eukaryotic cells, N6-methyladenosine (m^6^A) modification is regarded as the most common internal epigenetic modification in messenger RNAs (mRNAs) and long noncoding RNAs (lncRNAs), playing an important part in RNA splicing, degradation, and translation [5]. m^6^A epigenetic modification, a dynamic reversible process in mammalian cells, is regulated by m^6^A methylation regulators composed of methyltransferases (“writers”), demethylases (“erasers”), and binding proteins (“readers”). The formation of m^6^A methylation is mediated by methyltransferases including METTL3/14/16, WTAP, IRMA, ZC3H13, RBM15, RBM15B, and PCIF1, while the methylation removal process is regulated by demethylases consisting of FTO and ALKBH3/5. RNA-binding proteins, including YTHDC1/2, YTHDF1/2/3, HNRNPA2B1, LRPPRC, FMR1 TRMT112, ZCCHC4, NUDT21, CPSF6, CBLL1, SETD2, SRSF3, SRSF10, XRN1, NXF1, PRRC2A, IGF2BP1/2/3, IGFBP3, and RBMX, play a vital role in human cancer by binding m^6^A motif [6]. It has been reported that aberrant expression of m^6^A methylation regulators plays an important role in tumor occurrence, progression, and prognosis of certain cancers [7, 8]. For example, FTO, METTL3, and YTHDF1 could promote the progression and metastasis of cervical cancer and are potential biomarkers for the prognosis of cervical cancer [9, 10].

lncRNAs, a type of RNAs with transcript lengths over 200 nucleotides, constitute the largest group of noncoding RNAs in mammals and regulate about 70% of gene expression through interactions with DNA, RNA, and proteins [11]. In recent years, with the popularization of functional genomics research, the role of lncRNAs in tumor has become a new research hotspot. Aberrant lncRNA expression is related to tumor cell growth, invasion, and metastasis, thereby affecting the prognosis of tumors [12]. lncRNA GAS5-AS1 has been reported to suppress the tumorigenicity and metastasis of cervical cancer through increasing tumor suppressor GAS5 stability via interacting with ALKBH5 and decreasing GAS5 m^6^A modification [13]. High expression of lncRNA KCNMB2-AS1 is positively correlated with poor prognosis of cervical cancer by upregulating the oncogene IGF2BP3, which in turn increases the stability of KCNMB2-AS1 [14]; lncRNA ZFAS1 promotes the metastasis of cervical cancer by suppressing miR-647 mediated by METLL3 [15]. Some m^6^A-related lncRNA prognostic signatures associated with immune infiltration have been identified in gastric, colon, and lung cancers [16–18]. However, the full role of m^6^A-related lncRNAs in the prognosis and immune infiltration of cervical cancer remains unclear.

In this study, we identified m^6^A-related prognostic lncRNAs based on TCGA-cervical cancer database and then constructed and validated a prognostic model for cervical cancer patients. Subsequently, we assessed the correlation between consensus clustering of m^6^A-related prognostic lncRNAs, m^6^A-related lncRNA risk score, immune cell infiltration, and transforming growth factor (TGF) beta expression. In addition, we explored the difference in pathways between clustering subtypes and between risk subgroups.

## 2. Materials and Methods

### 2.1. Acquisition of Transcriptional and Clinical Data

The RNA sequencing data (FPKM type) of 306 cervical cancer samples and 3 normal tissues were downloaded from The Cancer Genome Atlas (TCGA, https://portal.gdc.cancer.gov/) database. The corresponding clinical data such as age, grade, Federation of Gynecology and Obstetrics (FIGO) stage, survival status, overall survival (OS), progression-free survival (PFS), and disease-specific survival (DSS) were obtained from UCSC Xena (https://xenabrowser.net/). All data were downloaded on June 7, 2021. A total of 273 cervical cancer patients were enrolled into the survival analysis based on the following inclusion criteria. Inclusion criteria were as follows: (1) pathologically confirmed cervical cancer, (2) available information on OS time and survival status, and (3) OS time > 30 days. The 273 patients were further randomly assigned into a training set (137 patients) and a validation set (136 patients) at a 1 : 1 ratio through using the caret package.

### 2.2. Identification of m^6^A-Related Prognostic lncRNAs

We extracted lncRNA and mRNA expression data according to the human genome annotation data. Pearson correlation coefficients between the expression levels of 36 m^6^A methylation regulators (METTL3/14/16, WTAP, IRMA, ZC3H13, RBM15, RBM15B, PCIF1, FTO, ALKBH3/5, YTHDC1/2, YTHDF1/2/3, HNRNPA2B1, LRPPRC, FMR1 TRMT112, ZCCHC4, NUDT21, CPSF6, CBLL1, SETD2, SRSF3, SRSF10, XRN1, NXF1, PRRC2A, IGF2BP1/2/3, IGFBP3, and RBMX) and lncRNAs were calculated to determine their coexpression relationship. The lncRNAs with ∣Pearson coefficient | >0.40 and *P* value < 0.001 were considered as m^6^A-related lncRNAs. Univariate Cox regression analysis was then performed to identify prognostic lncRNAs. The m^6^A-related lncRNAs with *P* value < 0.05 were regarded as m^6^A-related prognostic lncRNAs.

### 2.3. Bioinformatic Analysis

To functionally analyze the biological properties of m^6^A-related prognostic lncRNAs in cervical cancer, we utilized the “ConsensusClusterPlus” package (http://www.bioconductor.org/, resampling rate of 80% and 1000 iterations) to divide patients into different clustering subtypes. Gene set variation analysis (GSVA) was performed to explore the difference of pathways between different subgroups by using the “GSVA” package. The “c2.cp.kegg.v7.4.symbols” gene sets were downloaded from MSigDB database for running GSVA. Adjusted *P* value < 0.05 was regarded as significant. Single-sample gene-set enrichment analysis (ssGSEA) algorithm was used to quantify the relative infiltration levels of tumor microenvironment immune cells. The gene set for marking 23 immune cell types was acquired from the published study [19]. Least absolute shrinkage and selection operator (LASSO) regression model was used to construct a prognostic risk model through selecting the optimal penalty parameter associated with the minimum 10-fold cross-validation in the training set. The coefficients obtained from the LASSO regression model were used to yield the following risk score formula:

Risk score = ∑_*i*=1_^*n*^Coef_*i*_∗Exp_*i*_, 

where Coef_*i*_ is the coefficient and Exp_*i*_ means the expression of m^6^A-related lncRNAs. The risk score of each patient was calculated based on this equation. The median risk score in the training cohort was defined as a cutoff point to divide patients into the high- and low-risk groups, respectively.

### 2.4. Sample Collection

A total of 9 cervical cancer samples and 9 normal cervical tissue samples were collected by a gynecologic oncologist in the Gynecology Department of Shanxi Province Cancer Hospital in June 2021. Tumor tissues were taken from newly diagnosed patients with FIGO stage I/II cervical cancer who had not received any treatment. Normal cervical tissues were obtained from patients with hysteromyoma. Fresh tissue was placed into a tube containing 1 mL of RNAStore fluid (DP451, Tiangen Biotech Co., Ltd., Beijing, China) within 1 minute after leaving from the human body. The tube was then stored at 4°C for 24 hours. Subsequently, the RNAStore fluid was discarded and the tissue was frozen with liquid nitrogen and then stored at -80°C. All participants provided their written informed consent, and ethical approval was obtained from the Shanxi Province Cancer Hospital Science Research Ethics Committee (No. SJJ202105).

### 2.5. Quantitative Real-Time Polymerase Chain Reaction

To validate the expression levels of m^6^A-related prognostic lncRNAs in tumor samples and normal tissues, we performed quantitative real-time polymerase chain reaction (qRT-PCR) assay. Total RNA were isolated from 18 samples using RNA TRIzol reagent (Tiangen Biotech Co., Ltd., Beijing, China, #DP451). Then, cDNA synthesis was performed by using PrimeScript™ RT Master Mix (Takara Biomedical Technology Co., Ltd., Beijing, China, #RR036Q). Real-time PCR was conducted with TB Green Premix Ex Taq (Takara Biomedical Technology Co., Ltd., Beijing, China, #RR820A). Relative expression of lncRNAs was normalized to GAPDH and calculated by using 2-*ΔΔ*Ct method. Primer sequences used in our study are as follows: RPP38-DT-F: 5′-CCATCGGAGTCGCTGCAAAGTC-3′, RPP38-DT-R: 5′-AGGAGGAGGCTCATTAGGTCAGAAG-3′; AC024270.4-F: 5′-TCATGAGCCACGAAGTCAAGC-3′, AC024270.4-R: 5′-AGCCTTAAGTCTCAGGTCCTC-3′; AC008124.1-F: 5′-TGCCAACGACTTCTACCACCT-3′, AC008124.1-R: 5′-AGTCACCTCAGCTTTCCGTTC-3′; and AC025176.1-F: 5′-CTTCAACTGGCTTCCTTGCTT-3′, AC025176.1-R: 5′-ACAGGAAACTCCTTCGTCACA-3′.

### 2.6. Statistical Analysis

Statistical tests were conducted by R version 4.0.4. Wilcoxon rank sum test was applied to compare the quantitative data such as m^6^A-related prognostic lncRNAs, TGFbeta, and risk score between subgroups. Chi-square test was used to examine the difference between the training set and the validation set. The Kaplan-Meier method was used to draw survival curves, and log-rank test was performed to compare the survival difference between groups. Pearson correlation test was used for assessing the correlations between m^6^A-related prognostic lncRNAs and TGFbeta. The predictive accuracy of m^6^A-related lncRNA risk score for 3- and 5-year OS was evaluated using the receiver operating characteristic (ROC) curves and the area under the ROC curve (AUC). Univariate and multivariate Cox regression models were then used to analyze and validate the independent prognostic value of m^6^A-related lncRNA risk score.

## 3. Results

### 3.1. Identification of m^6^A-Related lncRNAs in Cervical Cancer

We extracted the expression levels of 36 m^6^A RNA methylation regulatory genes and a total of 14086 lncRNAs from TCGA-cervical cancer dataset. Of the 14086 lncRNAs, 116 positively or negatively correlated with m^6^A regulatory genes (∣Pearson correlation coefficient | >0.40 and *P* value < 0.001) were regarded as m^6^A-related lncRNAs. Among the 116 m^6^A-related lncRNAs, 7 were associated with the survival of patients and were considered as m^6^A-related prognostic lncRNAs (*P* value < 0.05, Table 1). The analysis results showed that AC024270.4, AC025176.1, AC008124.1, AL109811.2, and RPP38-DT were positively associated with OS and may be protective factors for the prognosis of patients with HR < 1. Nevertheless, AC015922.2 and AC099850.4 were inversely related to OS and might be risky prognostic biomarkers with HR > 1. Thus, these 7 m^6^A-related prognostic lncRNAs may play an important role in the prognosis of cervical cancer.

### 3.2. Expression of m^6^A-Related Prognostic lncRNAs in Cervical Cancer

**Figure 1:**
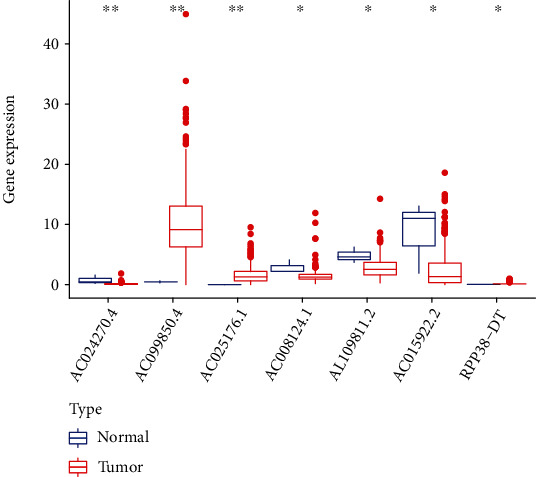
Expression profiles of 7 m^6^A-related prognostic lncRNAs between cervical cancer samples and normal tissues (^∗^*P* < 0.05; ^∗∗^*P* < 0.01). m^6^A: N6-methyladenosine; lncRNAs: long noncoding RNAs.

We compared the expression profiles of 7 m^6^A-related prognostic lncRNAs between cervical cancer samples and normal tissues to explore their potential biological function in the occurrence of cervical cancer. The expression levels of AC099850.4, RPP38-DT, and AC025176.1 in tumor samples were remarkably higher compared with normal tissues, while the levels of AC024270.4, AC008124.1, AL109811.2, and AC015922.2 in normal tissues were significantly higher than those in cancer samples (Figure 1). These results indicated that the 7 m^6^A-related prognostic lncRNAs may also possess potential biological roles in the initiation of cervical cancer.

### 3.3. Consensus Clustering for m^6^A-Related Prognostic lncRNAs and Association with Survival and Clinical Characteristics of Cervical Cancer Patients

**Figure 2:**
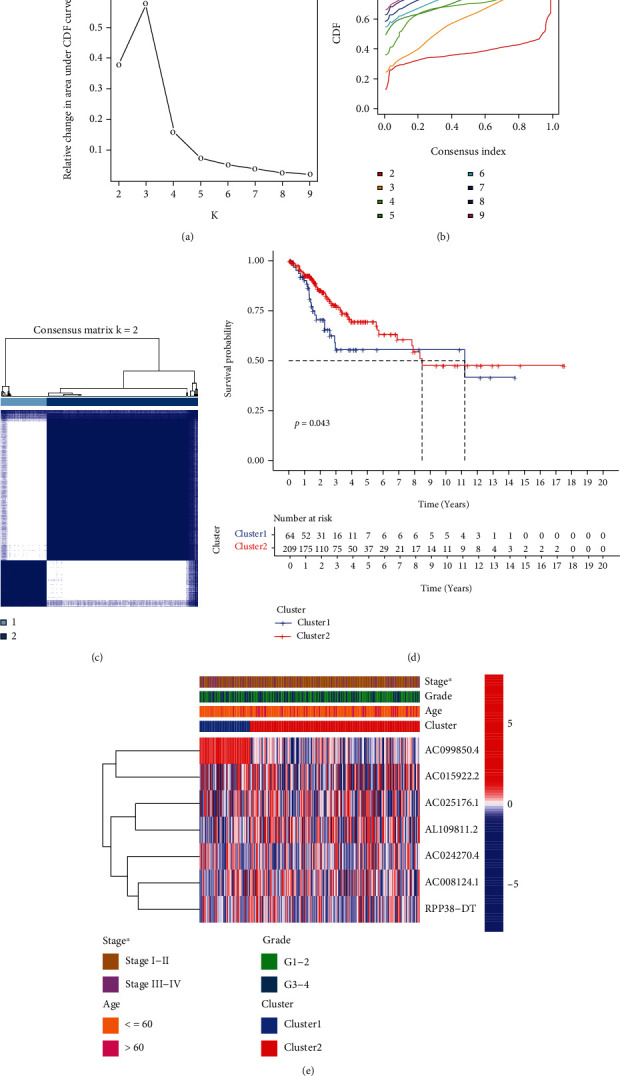
Different survival and clinicopathological characteristics in cluster 1/2 subtypes constructed based on 7 m^6^A-related prognostic lncRNAs. (a) CDF for *k* = 2 to 9. (b) Relative change in area under the CDF curve for *k* = 2 to 9. (c) Consensus clustering matrix for *k* = 2. (d) Kaplan-Meier curves of OS in cluster 1/2. (e) Heat map and clinicopathological features in cluster 1/2 (^∗^*P* < 0.05). m^6^A: N6-methyladenosine; lncRNAs: long noncoding RNAs; CDF: cumulative distribution function; OS: overall survival.

We constructed a consensus cluster consisting of 7 m^6^A-related prognostic lncRNAs through using the “ConsensusClusterPlus” package. The cumulative distribution function (CDF) and the area under the CDF curve of consensus clustering varied from *k* = 2 to 9 as shown in Figures 2(a) and 2(b). The clustering stability of *k* = 2 was optimal between *k* = 2 and 9. A total of 273 cervical cancer patients were clustered into two clustering subtypes: cluster 2 (*n* = 209) and cluster 1 (*n* = 64) (Figure 2(c)). Furthermore, we compared the OS between two clusters and found that patients in cluster 2 had notably longer OS than patients in cluster 1 (*P* value = 0.043, Figure 2(d)). Furthermore, we explored the association between clustering subgroups and clinicopathological features of patients (Figure 2(e)). Cluster 1 had significantly more patients with advanced stage than cluster 2. However, no significant difference was observed in age and pathological grade between the two clustering subtypes.

### 3.4. Association of TGFbeta with m6A-Related Prognostic lncRNAs

**Figure 3:**
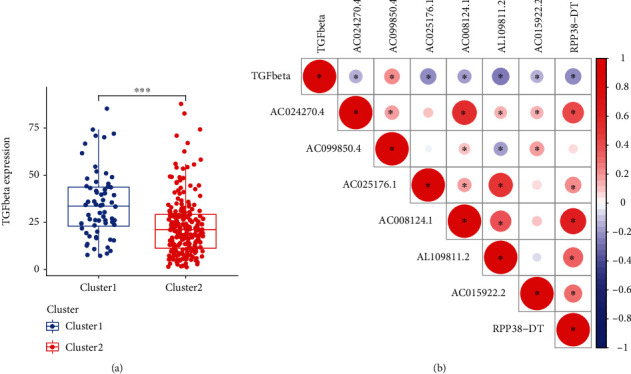
Associations of TGFbeta with m^6^A-related prognostic lncRNAs. (a) Expression of TGFbeta in cluster 1/2 subtypes (^∗∗∗^*P* < 0.001). (b) Relationship between TGFbeta and m^6^A-related prognostic lncRNAs. m^6^A: N6-methyladenosine; lncRNAs: long noncoding RNAs.

To explore the involvement of immune suppressor TGFbeta with m^6^A-related prognostic lncRNAs, we assessed its expression levels in two clustering subtypes and its correlation with 7 m^6^A-related prognostic lncRNAs. As expected, the expression level of TGFbeta was markedly higher in cluster 1 with poor prognosis compared with cluster 2 (Figure 3(a)). Additionally, TGFbeta was positively correlated with AC099850.4, but negatively associated with AC024270.4, AC025176.1, AC008124.1, AL109811.2, AC015922.2, AC000068.1, and RPP38-DT (Figure 3(b)). Most of the 7 m^6^A-related prognostic lncRNAs were positively correlated except that AC099850.4 was inversely related to AL109811.2.

### 3.5. Immune Cell Infiltration and Pathway Enrichment Analyses in Different Consensus Clustering Subtypes

**Figure 4:**
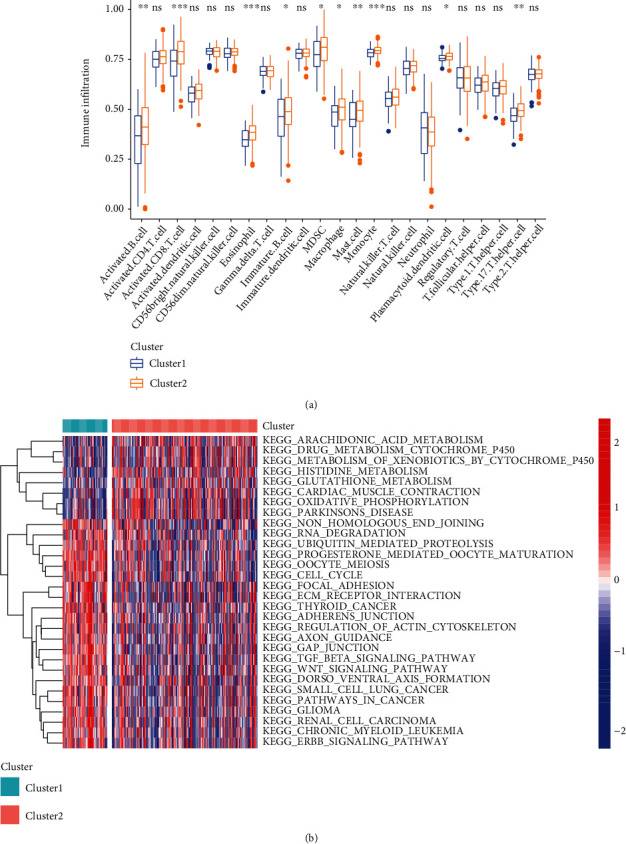
Tumor immune microenvironment characteristics in cluster 1/2 subtypes. (a) The abundance of immune infiltrating cells (^∗^*P* < 0.05; ^∗∗^*P* < 0.01; ^∗∗∗^*P* < 0.001). (b) Heat map and the activation states of biological pathways. m^6^A: N6-methyladenosine; lncRNAs: long noncoding RNAs.

To explore the association of m^6^A-related prognostic lncRNAs with tumor immune microenvironment, we compared the immune cell infiltrates between two clustering subtypes. Cluster 2 with better prognosis exhibited higher levels of immune cells such as activated B cell, activated CD8 T cell, and eosinophils (Figure 4(a)). Next, we performed GSVA to explore the biological behaviors of two clustering subtypes. Cluster 2 was associated with hallmark pathways such as cardiac muscle contraction, arachidonic acid metabolism, oxidative phosphorylation, and histidine catabolism (Figure 4(b)). Cluster 1 was significantly enriched in pathways such as gap junction, focal adhesion, TGFbeta signaling pathway, Wnt signaling pathway, ubiquitin-mediated proteolysis, cell cycle, and pathways in cancer. These results indicated that the two clustering subtypes might have markedly different immune landscape.

### 3.6. Establishment of m^6^A-Related lncRNA Risk Score

**Figure 5:**
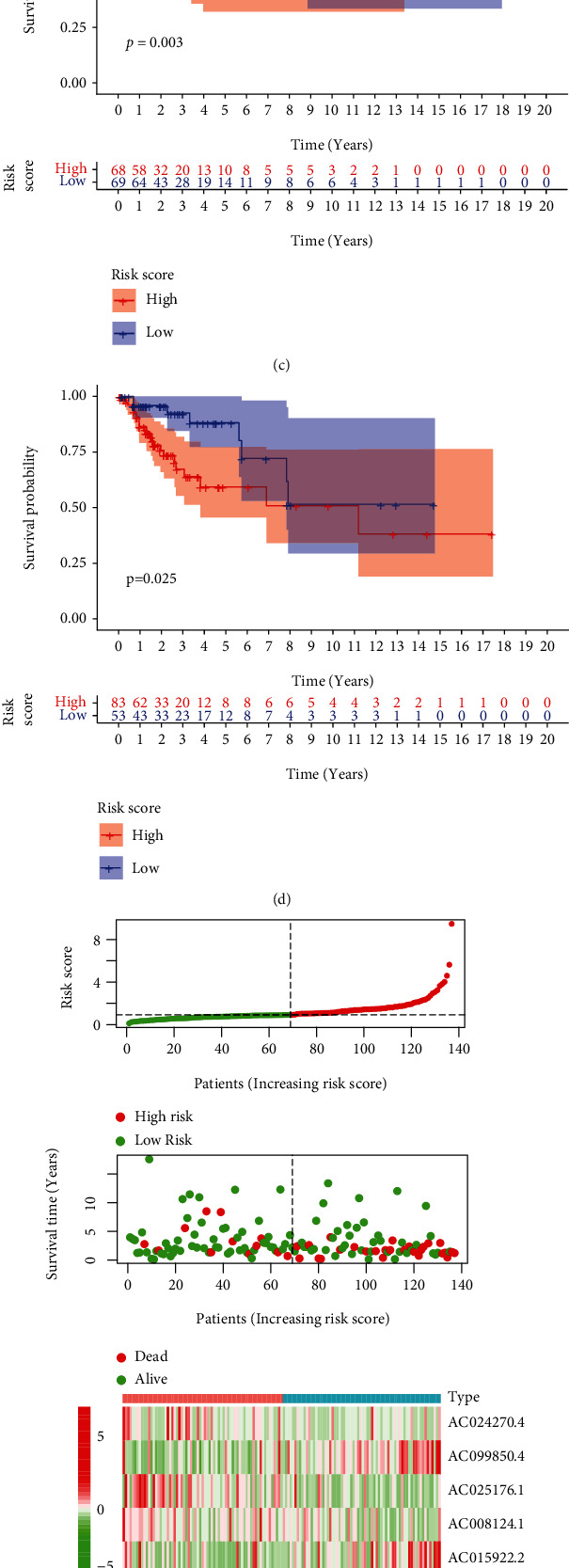
Establishment of m^6^A-related lncRNA risk score. (a) Partial likelihood deviance for tuning the parameter selection in LASSO regression model. (b) LASSO coefficient profiles. Kaplan-Meier curves of OS in the (c) training cohort and (d) validation cohort. Distribution of risk score, OS, and survival status and heat map of risk score in the (e) training set and (f) validation set. m^6^A: N6-methyladenosine; lncRNA: long noncoding RNA; LASSO: least absolute shrinkage and selection operator; OS: overall survival.

**Figure 6:**
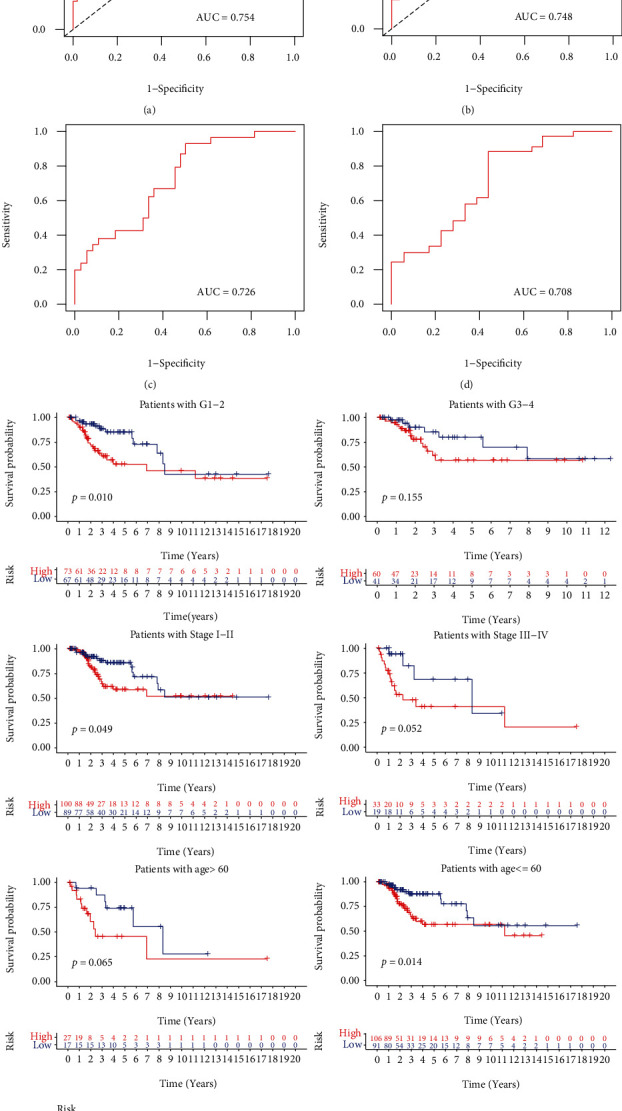
The ROC curves and stratified survival Kaplan-Meier curves of m^6^A-related lncRNA risk score in OS prediction. The (a) 3-year and (b) 5-year ROC curves in the training set. The (c) 3-year and (d) 5-year ROC curves in the validation set. (e) Stratified survival Kaplan-Meier curves. m^6^A: N6-methyladenosine; lncRNAs: long noncoding RNAs; OS: overall survival; ROC: receiver operating characteristic.

We further explored the role of 7 m^6^A-related prognostic lncRNAs in the OS by using LASSO regression model. The baseline characteristics, including age, grade, and FIGO stage, were not significantly different between the training and validation sets (all *P* values > 0.05 Supplementary Table 1). A risk score model was constructed in the training set. As shown in Figures 5(a) and 5(b), 6 m^6^A-related lncRNAs were identified according to the minimum lambda criteria. The risk score of each patient was calculated using the coefficients (Table 2) and expression levels of 6 m^6^A-related lncRNA signatures. Then, the median risk score in the training cohort was used as a cutoff point to divide patients into the high- and low-risk groups.

No matter in the training (*P* value = 0.003, Figure 5(c)) or validation (*P* value = 0.025, Figure 5(d)) sets, the high-risk group had worse OS than the low-risk group. The distribution of risk score, OS, and expression profiles of 6 m^6^A-related lncRNA signatures in the training and validation sets is displayed in Figures 5(e) and 5(f), respectively. The OS in the low-risk group was evidently longer than that in the high-risk group. The heat map results demonstrated that the expression levels of AC099850.4 and AC015922.2 were upregulated in the high-risk group as prognostic risky factors, while AC024270.4, AC008124.1, AC025176.1, and RPP38-DT were downregulated in the high-risk group as prognostic protective factors. In order to assess the prognostic accuracy of risk score, we performed 3- and 5-year ROC analyses and found that the 3-year and 5-year AUC values in the training group were 0.754 (Figure 6(a)) and 0.748 (Figure 6(b)), respectively; in the validation group, the 3-year and 5-year AUC values were 0.726 (Figure 6(c)) and 0.708 (Figure 6(d)). These AUC values indicated that m^6^A-related lncRNA risk score had a good discrimination performance in cervical cancer prognosis. Further stratified survival analysis results displayed that the OS time in the low-risk group was significantly longer than that in the high-risk group, no matter for patients with age ≤ 60 years, grade 1/2, or stage I/II (Figure 6(e)). Although no significant survival difference was displayed between the high-risk group and the low-risk group in patients with age > 60 years, grade 3/4, or stage III/IV, a better prognostic tendency was observed in the high-risk group.

### 3.7. Independent Prognostic Value of m^6^A-Related lncRNA Risk Score in the OS of Cervical Cancer Patients

**Figure 7:**
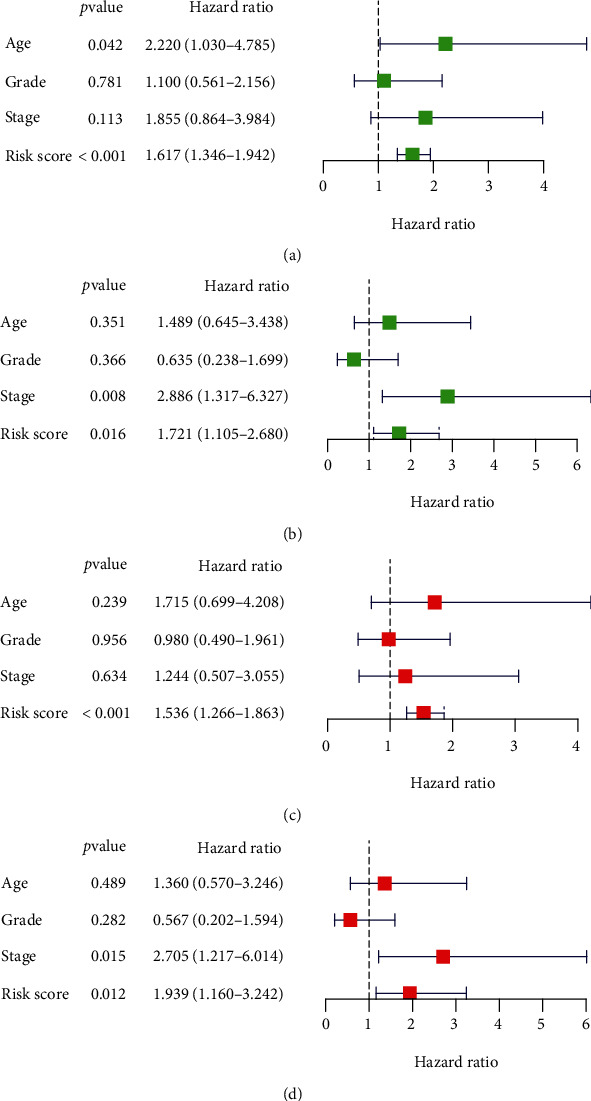
Univariate and multivariate Cox regression analyses of cervical cancer patients based on risk score and clinicopathological features. Univariate Cox regression analyses in the (a) training cohort and in the (b) validation cohort. Multivariate Cox regression analyses in the (c) training cohort and in the (d) validation cohort.

In order to ascertain whether m^6^A-related lncRNA risk score was an independent predictor for the OS in cervical cancer, we performed univariate and multivariate Cox regression analyses in the training and validation groups, respectively. In the training cohort, univariate Cox analysis results showed that age over 60 years and high-risk score were risk factors for the prognosis of cervical cancer patients (Figure 7(a)). In the validation cohort, univariate Cox analysis results displayed that advanced stage and high-risk score were risk factors for cervical cancer prognosis (Figure 7(b)). In the multivariate Cox analysis, risk score was still a risky factor for poor prognosis in both the training cohort (HR = 1.54, *P* value < 0.001, Figure 7(c)) and the validation set (HR = 1.94, *P* value = 0.012, Figure 7(d)). Our analysis results suggested that m^6^A-related lncRNA risk score was an independent predictor for the OS in cervical cancer patients.

### 3.8. Validation of Independent Prognostic Value of m^6^A-Related lncRNA Risk Score

**Figure 8:**
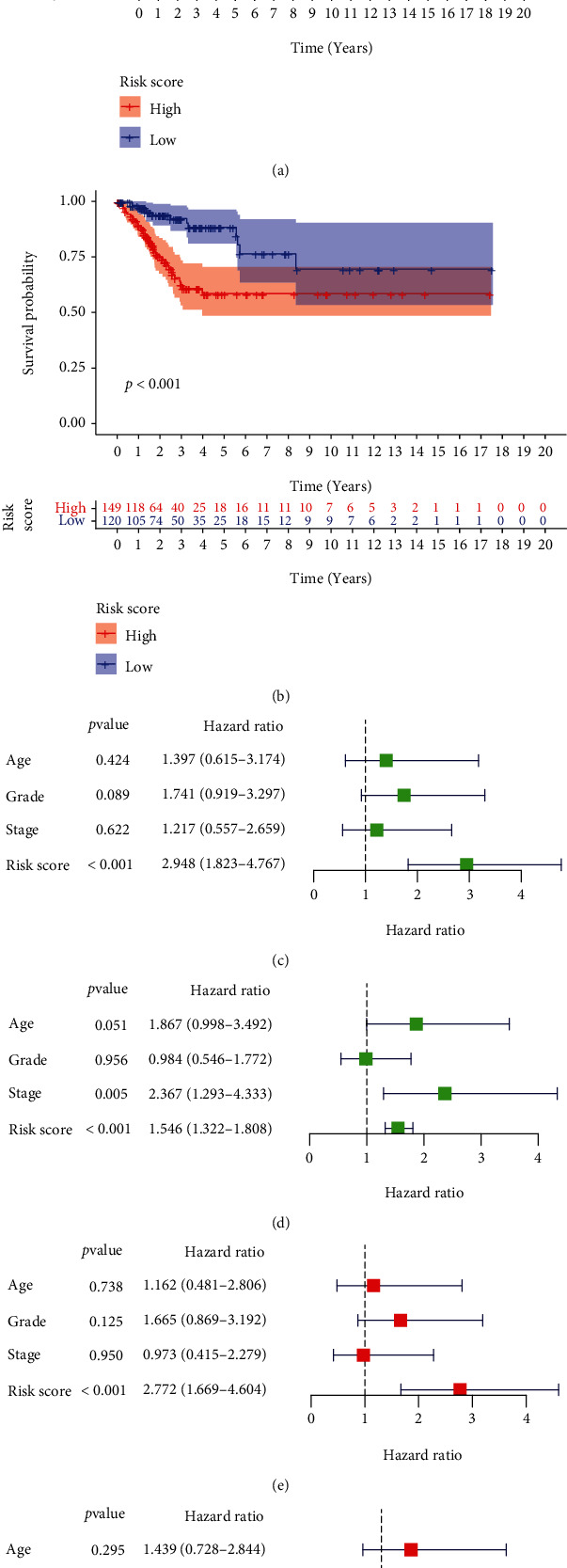
The independent prognostic value of m^6^A-related lncRNA risk score in the DSS and PFS. (a) Kaplan-Meier curves of PFS. (b) Kaplan-Meier curves of DSS. Univariate Cox regression analyses in the (c) PFS and (d) DSS. Multivariate Cox regression analyses in the (e) PFS and (f) DSS. m^6^A: N6-methyladenosine; lncRNA: long noncoding RNA; DSS: disease-specific survival; PFS: progression-free survival.

In view of the significance of PFS and DSS in tumor prognosis, we further explored the prognostic value of m^6^A-related lncRNA risk score in the PFS and DSS of cervical cancer patients. Patients with high-risk score had dramatically worse PFS (*P* value = 0.002, Figure 8(a)) and DSS (*P* value < 0.001, Figure 8(b)) in comparison with those with low-risk score. No matter in the univariate (Figures 8(c) and 8(d)) or multivariate (Figures 8(e) and 8(f)) Cox analyses, risk score was significantly associated with PFS and DSS. The progression risk in patients with high-risk score was 2.77 times as high as that in patients with low-risk score. The risk of cervical cancer-specific death in patients with high-risk score was 1.46 times higher than that in patients with low-risk score. The above results indicated again that m^6^A-related lncRNA risk score had good prognostic value in cervical cancer.

### 3.9. Correlation of m^6^A-Related lncRNA Risk Score with Clustering Subtypes, Clinical Characteristics, and TGFbeta Expression of Cervical Cancer Patients

**Figure 9:**
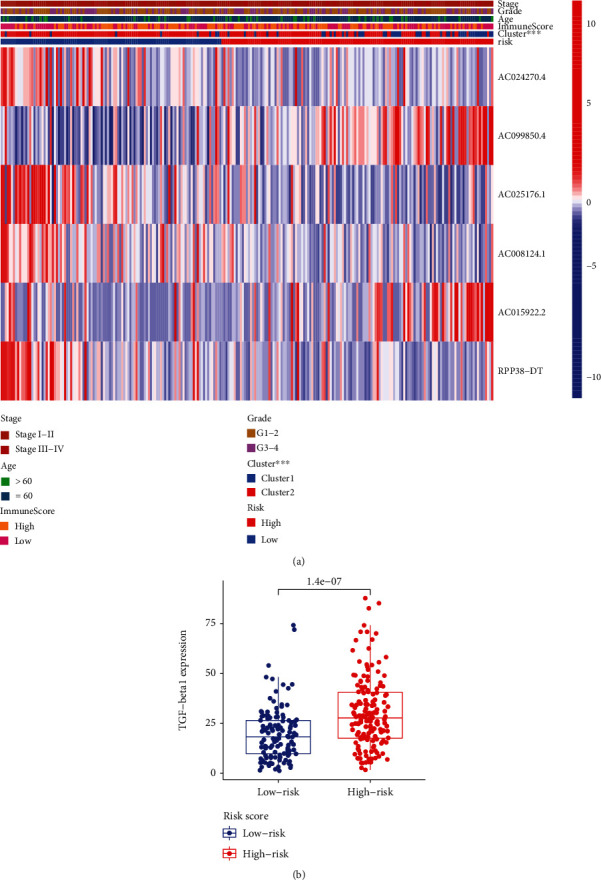
Correlation of risk score with clustering subtypes, clinical characteristics, and TGFbeta expression. (a) Correlation of risk score with clustering subtypes and clinical characteristics. (b) Expression of TGFbeta in the high- and low-risk groups.

Subsequently, we compared the difference in consensus clustering, clinical characteristics, and TGFbeta expression between the high-risk and low-risk groups. As shown in Figure 9(a), the high-risk group was preferentially associated with cluster 1. However, no significant difference in age, stage, and grade was observed between the high-risk and low-risk groups. In addition, we found that the expression level of TGFbeta of the high-risk group was markedly higher compared with that of the low-risk group Figure 9(b).

### 3.10. Immune Cell Infiltration and Pathway Enrichment Analyses in the High-Risk and Low-Risk Groups

**Figure 10:**
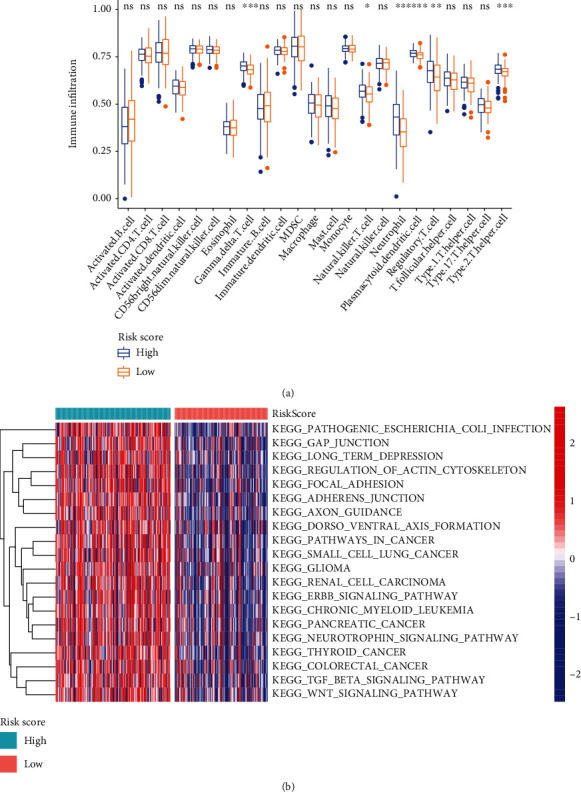
Tumor immune microenvironment characteristics in the high- and low-risk groups. (a) The abundance of immune infiltrating cells (^∗^*P* < 0.05; ^∗∗^*P* < 0.01; ^∗∗∗^*P* < 0.001). (b) Heat map and the activation states of biological pathways.

To explore the immune infiltration and biological behaviors of m^6^A-related lncRNA risk score, we performed ssGSEA and GSVA in the high-risk and low-risk groups. The ssGSEA results showed that the high-risk group had markedly higher regulatory T cell expression relative to the low-risk group (Figure 10(a)). The GSVA results displayed that the high-risk group was characterized by enrichment of virus-associated, stromal, and malignant pathways such as pathogenic *Escherichia coli* infection, gap junction, focal adhesion, ErbB signaling pathway, TGFbeta signaling pathway, Wnt signaling pathway, and pathways in cancer (Figure 10(b)). The above results indicated again that the 6 m^6^A-related lncRNAs used in calculating risk score were involved in the malignant process and immune cell infiltration of cervical cancer.

### 3.11. Validation of the Expression Levels of Four m6A-Related lncRNAs in Cervical Cancer Samples

**Figure 11:**
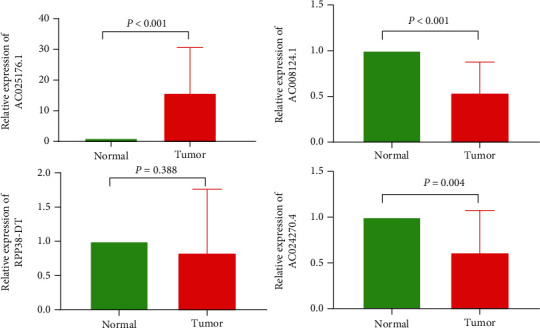
Relative expression levels of m^6^A-related prognostic lncRNAs in cervical cancer tissues and normal cervical tissues.

To validate the expression levels of m^6^A-related lncRNAs in cervical cancer samples, we detected four m^6^A-related prognostic lncRNAs in 9 tumor tissues and 9 normal samples by using qRT-PCR assay. Our results showed that the expression levels of AC024270.4 and AC008124.1 were relatively lower in cervical cancer samples than normal tissues, while AC025176.1 was upregulated in tumor tissues (Figure 11). No significant difference was observed in RPP38-DT expression between two groups.

## 4. Discussion

Cervical cancer ranks fourth in cancer-related deaths in women globally, posing a serious threat to female health [1]. It is urgent to find new biomarkers to better predict the prognosis of cervical cancer and develop personalized treatment plans. Some m^6^A methylation regulators and lncRNAs have been reported to be closely associated with the progression of cervical cancer [9, 10, 13–15]. However, hitherto, no study has sought to construct a prognostic model based on m^6^A-related lncRNAs. In this study, we explored the predictive value of m^6^A-related lncRNAs in the prognosis of cervical cancer by combination of these two perspectives. In addition, we explored the association of m^6^A-related lncRNAs with tumor microenvironment immune infiltration. Finally, qRT-PCR was used to detect the expression levels of four m^6^A-related prognostic lncRNAs between cervical cancer tissues and normal tissues.

Based on TCGA-cervical cancer data, we identified 7 m^6^A-related prognostic lncRNAs by using Pearson correlation analysis and univariate Cox regression analysis. All the 7 m^6^A-related lncRNAs had significantly different expression levels between cervical cancer samples and normal tissues. Then, we divided cervical cancer patients into two clustering subtypes (cluster 1 and cluster 2) according to the expression of 7 m^6^A-related prognostic lncRNAs. As expected, cluster 1 with poor prognosis was preferentially associated with advanced clinical stage and had higher TGFbeta relative to cluster 2. TGFbeta was a tumor suppressor at the early stage of carcinogenesis inducing apoptosis of premalignant cells, while it acts as a tumor promoter at later stage, promoting tumor growth and metastasis by stimulating epithelial-mesenchymal transition, tumor angiogenesis, and cancer-associated fibroblasts [20]. TGFbeta, the most prominent immunosuppressive cytokine in tumor microenvironment, also plays an important role in tumor immune evasion and poor response to antitumor immunotherapy by inhibiting the generation and function of effector immune cells and promoting the expansion of regulatory T cells [21]. Elevated TGFbeta promotes the growth, invasion, and migration of cervical cancer cells, suggesting poor prognosis [22]. In addition, we found that TGFbeta was positively correlated with potential risky prognostic factor AC099850.4, but negatively correlated with potential protective prognostic factors AC024270.4, AC025176.1, AC008124.1, AL109811.2, and RPP38-DT. Tumor immune microenvironment is complex and heterogeneous, which affects tumor progression and therapeutic effect [23, 24]. Cluster 2 with better prognosis exhibited higher levels of effector immune cells such as activated B cell and activated CD8 T cell, as well as enrichment of pathways such as arachidonic acid metabolism, oxidative phosphorylation, and histidine catabolism. Mediators released from the arachidonic acid metabolic pathway play a vital role in maintaining normal function of the immune system [25]. Mitochondrial oxidative phosphorylation plays important roles in maintaining the proliferation of T cells and inhibiting T cell exhaustion [26]. Cluster 1 was associated with immunosuppressive and tumorigenic activation pathways such as TGFbeta signaling pathway, Wnt signaling pathway, ubiquitin-mediated proteolysis, and pathways in cancer. TGFbeta signaling inhibits the normal function of immunity system, promoting tumor grow, metastasis, and immune evasion [21, 27]. Wnt signaling promotes tumor immune escape and limits antitumor immunotherapy response in several cancers including cervical cancer [28]. Ubiquitin-mediated proteolysis is closely associated with multiple biological processes including cell growth, differentiation, immune regulation, and inflammatory response [29]. These results indicated that m^6^A-related lncRNAs were involved in the prognosis and immune cell infiltration of cervical cancer patients.

Subsequently, we identified 6 out of 7 m6A-related prognostic lncRNAs to construct a risk score model for predicting the outcomes of patients. Whether in the training set or in the validation set, patients in the high-risk group had shorter OS time in comparison with those in the low-risk group. Consistent with results from univariate Cox regression analysis, AC024270.4, AC008124.1, AC025176.1, and RPP38-DT were protective biomarkers for the prognosis of cervical cancer, while AC015922.2 and AC099850.4 were risky biomarkers. Meanwhile, the m^6^A-related risk score was an independent prognostic factor of OS in cervical cancer patients. We also validated the independent prognostic value of m^6^A-related risk score in the PFS and DSS. In brief, the m^6^A-related risk score can accurately predict the prognosis of patients with cervical cancer. Previous studies about the 6 m^6^A-related lncRNAs were few. AC015922.2 was a prognostic risk marker in our study, but a prognostic protective biomarker in clear cell renal cell carcinoma [30]. AC099850.4 may be involved in the progression of ovarian cancer through lncRNA-miRNA-mRNA competing triplets [31]. More research about the 6 m^6^A-related lncRNAs is needed in the future.

Next, we found the high-risk group had significantly higher TGFbeta levels than the low-risk group. TGFbeta is an important immunosuppressive factor, predicting poor prognosis of cervical cancer [32]. In view of the vital roles of tumor immune microenvironment in tumor prognosis [33], we compared immune cell infiltration between two different risk score groups. The high-risk group had higher abundance of regulatory T cell, a type of immunosuppressive T cell, which can be expanded by TGFbeta [21]. Additionally, the high-risk group was associated with pathways such as TGFbeta signaling pathway, Wnt signaling pathway, ErbB signaling pathway, and pathways in cancer. It is known that TGFbeta signaling and Wnt signaling can promote tumor immune evasion and metastasis and are potential antitumor therapeutic targets [34, 35]. ErbB signaling and Wnt signaling play a carcinogenic role and promote tumor progression in many cancer types [35, 36]. Consistent with the prognostic protective role of AC024270.4 and AC008124.1, their expression levels in cervical cancer were significantly lower than those in normal cervix. However, AC025176.1 was markedly upregulated in tumor tissues than in normal tissues. We speculate that AC025176.1 might play different roles in premalignant cells and cancer cells of cervical cancer.

Despite some positive results, there are still some limitations in our study. First, our analysis results were based on TCGA database. More clinical cohorts should be used to validate the prognostic value of identified m^6^A-related lncRNAs. Second, we only performed preliminary expression and mechanism studies on m^6^A-related prognostic lncRNAs. Their full biological mechanism remains to be further explored using in vitro and in vivo experiments.

## 5. Conclusions

In summary, our study systematically analyzed the expression, prognostic value, and impact on immune infiltration of m^6^A-related lncRNAs in cervical cancer. These findings provided some clues for future studies about m^6^A-related lncRNAs as promising therapeutic targets for cervical cancer.

## Figures and Tables

**Table 1:** Identification of m^6^A-related prognostic lncRNAs in cervical cancer.

m^6^A-related prognostic lncRNAs	HR (95% CI)	*P* value
AC024270.4	0.048 (0.004, 0.581)	0.017
AC099850.4	1.042 (1.007, 1.079)	0.018
AC025176.1	0.806 (0.659, 0.984)	0.034
AC008124.1	0.628 (0.422, 0.935)	0.022
AL109811.2	0.801 (0.672, 0.954)	0.013
AC015922.2	1.088 (1.022, 1.159)	0.009
RPP38-DT	0.068 (0.005, 0.838)	0.036

m^6^A: N6-methyladenosine; lncRNAs: long noncoding RNAs; HR: hazard ratio; CI: confidence interval.

**Table 2:** Coefficients of 6 m^6^A-related lncRNA signatures.

Gene	Coefficients
AC024270.4	-0.896960755
AC099850.4	0.046185879
AC025176.1	-0.167161103
AC008124.1	-0.064495077
AC015922.2	0.101961902
RPP38-DT	-1.770669581

## Data Availability

The data and codes used to support the findings of this study are available from the corresponding author upon request.

## Supplementary Materials

Supplementary Materials Supplementary Table 1: clinicopathological features of patients in the training and validation sets.

## References

[1] Sung H., Ferlay J., Siegel R. L. (2021). Global cancer statistics 2020: GLOBOCAN estimates of incidence and mortality worldwide for 36 cancers in 185 countries. *CA: a Cancer Journal for Clinicians*.

[2] Cao W., Chen H. D., Yu Y. W., Li N., Chen W. Q. (2021). Changing profiles of cancer burden worldwide and in China: a secondary analysis of the global cancer statistics 2020. *Chinese Medical Journal*.

[3] Cohen A. C., Roane B. M., Leath C. A. (2020). Novel therapeutics for recurrent cervical cancer: moving towards personalized therapy. *Drugs*.

[4] Marret G., Borcoman E., Le Tourneau C. (2019). Pembrolizumab for the treatment of cervical cancer. *Expert Opinion on Biological Therapy*.

[5] Haruehanroengra P., Zheng Y. Y., Zhou Y., Huang Y., Sheng J. (2020). RNA modifications and cancer. *RNA Biology*.

[6] Chen X. Y., Zhang J., Zhu J. S. (2019). The role of m6A RNA methylation in human cancer. *Molecular Cancer*.

[7] Tian S., Lai J., Yu T., Li Q., Chen Q. (2020). Regulation of gene expression associated with the N6-methyladenosine (m6A) enzyme system and its significance in cancer. *Frontiers in Oncology*.

[8] Yi Y. C., Chen X. Y., Zhang J., Zhu J. S. (2020). Novel insights into the interplay between m6A modification and noncoding RNAs in cancer. *Molecular Cancer*.

[9] Zou D., Dong L., Li C., Yin Z., Rao S., Zhou Q. (2019). The m6A eraser FTO facilitates proliferation and migration of human cervical cancer cells. *Cancer Cell International*.

[10] Wang Q., Guo X., Li L. (2020). N^6^-Methyladenosine METTL3 promotes cervical cancer tumorigenesis and Warburg effect through YTHDF1/HK2 modification. *Cell Death & Disease*.

[11] Bhan A., Soleimani M., Mandal S. S. (2017). Long noncoding RNA and cancer: a new paradigm. *Cancer Research*.

[12] Cáceres-Durán M. Á., Ribeiro-Dos-Santos Â., Vidal A. F. (2020). Roles and mechanisms of the long noncoding RNAs in cervical cancer. *International Journal of Molecular Sciences*.

[13] Wang X., Zhang J., Wang Y. (2019). Long noncoding RNA GAS5-AS1 suppresses growth and metastasis of cervical cancer by increasing GAS5 stability. *American Journal of Translational Research*.

[14] Zhang Y., Wang D., Wu D., Zhang D., Sun M. (2020). Long noncoding RNA KCNMB2-AS1 stabilized by N<sup>6</sup>-methyladenosine modification promotes cervical cancer growth through acting as a competing endogenous RNA. *Cell Transplantation*.

[15] Yang Z., Ma J., Han S., Li X., Guo H., Liu D. (2020). <p>ZFAS1 exerts an oncogenic role via suppressing miR-647 in an m<sup>6</sup>A-dependent manner in cervical cancer</p>. *Onco Targets and Therapy*.

[16] Wang H., Meng Q., Ma B. (2021). Characterization of the prognostic m6A-related lncRNA signature in gastric cancer. *Frontiers in Oncology*.

[17] Chai X. K., Qi W., Zou C. Y., He C. X., Su M., Zhao D. Q. (2021). Potential prognostic value of a seven m6A-related LncRNAs signature and the correlative immune infiltration in colon adenocarcinoma. *Frontiers in Genetics*.

[18] Weng C., Wang L., Liu G., Guan M., Lu L. (2021). Identification of a N6-methyladenosine (m6A)-related lncRNA signature for predicting the prognosis and immune landscape of lung squamous cell carcinoma. *Frontiers in Oncology*.

[19] Charoentong P., Finotello F., Angelova M. (2017). Pan-cancer immunogenomic analyses reveal genotype-immunophenotype relationships and predictors of response to checkpoint blockade. *Cell Reports*.

[20] Hao Y., Baker D., Ten Dijke P. (2019). TGF-*β*-mediated epithelial-mesenchymal transition and cancer metastasis. *International Journal of Molecular Sciences*.

[21] Batlle E., Massagué J. (2019). Transforming growth factor-*β* signaling in immunity and cancer. *Immunity*.

[22] Xiao L., Zhu H., Shu J., Gong D., Zheng D., Gao J. (2022). Overexpression of TGF-*β*1 and SDF-1 in cervical cancer-associated fibroblasts promotes cell growth, invasion and migration. *Archives of Gynecology and Obstetrics*.

[23] Anderson N. M., Simon M. C. (2020). The tumor microenvironment. *Current Biology*.

[24] Binnewies M., Roberts E. W., Kersten K. (2018). Understanding the tumor immune microenvironment (TIME) for effective therapy. *Nature Medicine*.

[25] Hanna V. S., Hafez E. A. A. (2018). Synopsis of arachidonic acid metabolism: a review. *Journal of Advanced Research*.

[26] Vardhana S. A., Hwee M. A., Berisa M. (2020). Impaired mitochondrial oxidative phosphorylation limits the self-renewal of T cells exposed to persistent antigen. *Nature Immunology*.

[27] Chung J. Y., Chan M. K., Li J. S. (2021). TGF-*β* signaling: from tissue fibrosis to tumor microenvironment. *International Journal of Molecular Sciences*.

[28] Patel S., Alam A., Pant R., Chattopadhyay S. (2019). Wnt signaling and its significance within the tumor microenvironment: novel therapeutic insights. *Frontiers in Immunology*.

[29] Ciechanover A., Orian A., Schwartz A. L. (2000). Ubiquitin-mediated proteolysis: biological regulation via destruction. *BioEssays*.

[30] Yang W., Zhou J., Zhang K. (2021). Identification and validation of the clinical roles of the VHL-related LncRNAs in clear cell renal cell carcinoma. *Journal of Cancer*.

[31] Zhao J., Song X., Xu T. (2020). Identification of potential prognostic competing triplets in high-grade serous ovarian cancer. *Frontiers in Genetics*.

[32] Lin H., Huang C. C., Ou Y. C. (2012). High immunohistochemical expression of TGF-*β*1 predicts a poor prognosis in cervical cancer patients who harbor enriched endoglin microvessel density. *International Journal of Gynecological Pathology*.

[33] Gasser S., Lim L. H. K., Cheung F. S. G. (2017). The role of the tumour microenvironment in immunotherapy. *Endocrine-Related Cancer*.

[34] Xie F., Ling L., van Dam H., Zhou F., Zhang L. (2018). TGF-*β* signaling in cancer metastasis. *Acta Biochim Biophys Sin Shanghai*.

[35] Parsons M. J., Tammela T., Dow L. E. (2021). WNT as a driver and dependency in cancer. *Cancer Discovery*.

[36] Zhao R., Song J., Jin Y., Liu Y. (2021). Long noncoding RNA HOXC-AS3 enhances the progression of cervical cancer via activating ErbB signaling pathway. *Journal of Molecular Histology*.

