# Supplementary material for: m6A-Related lncRNAs Are Potential Prognostic Biomarkers of Cervical Cancer and Affect Immune Infiltration
Source: Dis Markers. 2022 Apr 11;2022:8700372. doi: 10.1155/2022/8700372 (PMC9011170; doi:10.1155/2022/8700372)
Supplement: Supplementary Materials — Supplementary Table 1: clinicopathological features of patients in the training and validation sets. [file 8700372.f1.docx]

| **Supplementary Table 1. Clinicopathological features of patients in the training and validation sets** | | | | |
| --- | --- | --- | --- | --- |
| Variables | The training group | The validation group | χ*^2^* | *P* values |
|  | No (%) | No (%) |  |  |
| Age (years) |  |  | 0.12 | 0.756 |
| ≤ 60 | 113 (82.5) | 110 (80.9) |  |  |
| > 60 | 24 (17.5) | 26 (19.1) |  |  |
| Grade |  |  | 3.52 | 0.177 |
| Unknown | 13 (9.5) | 13 (9.6) |  |  |
| G1-2 | 64 (46.7) | 78 (57.4) |  |  |
| G3-4 | 60 (43.8) | 45 (33.1) |  |  |
| FIGO stage |  |  | 1.66 | 0.429 |
| Unknown | 2 (1.5) | 4 (2.9) |  |  |
| Stage I-II | 109 (79.6) | 100 (73.5) |  |  |
| Stage III-IV | 26 (19.0) | 32 (23.5) |  |  |
| FIGO, Federation of Gynecology and Obstetrics. | | | | |
